# The expression of TGF-β1, Smad3, phospho-Smad3 and Smad7 is correlated with the development and invasion of nonfunctioning pituitary adenomas

**DOI:** 10.1186/1479-5876-12-71

**Published:** 2014-03-18

**Authors:** Li Zhenye, Li Chuzhong, Wu Youtu, Lan Xiaolei, Cao Lei, Hong Lichuan, Wang Hongyun, Wu Yonggang, Wang Fei, Zhang Yazhuo

**Affiliations:** 1 Beijing Neurosurgical Institute, Capital Medical University, Beijing 100050, China

**Keywords:** Nonfunctioning pituitary adenoma, Invasion, TGF-β1, Smad2, Smad3, Smad4, Smad7

## Abstract

**Background:**

Transforming growth factor β (TGF-β) signaling functions as a suppressor or a promoter in tumor development, depending on the tumor stage and type. However, the role of TGF-β signaling in nonfunctioning pituitary adenomas (NFPAs) has not been explored.

**Methods:**

TGF-β1, Smad2, phospho-Smad2 (p-Smad2), Smad3, phospho-Smad3 (p-Smad3), Smad4, and Smad7 were detected in 5 cases of normal anterior pituitaries, 29 cases of invasive NFPAs, and 21 cases of noninvasive NFPAs by real-time quantitative reverse transcription polymerase chain reaction (qRT-PCR), Western blot, and immunohistochemical analysis.

**Results:**

The Smad3 and p-Smad3 protein levels gradually decreased from normal anterior pituitaries, noninvasive NFPAs, to invasive NFPAs. However, there were no significant differences in Smad2 (*P* = 0.122) and p-Smad2 protein levels (*P* = 0.101) or Smad2 mRNA level (*P* = 0.409). In addition, the TGF-β1 mRNA level gradually decreased while the Smad7 mRNA level gradually increased from normal anterior pituitaries, noninvasive NFPAs, to invasive NFPAs. Furthermore, proliferating cell nuclear antigen (PCNA) mRNA level was markedly increased in invasive NFPAs compared to noninvasive ones (*P* < 0.01), and its level was negatively correlated with Smad3 mRNA level (*P* < 0.01).

**Conclusion:**

The activity of TGF-β signaling may be restrained in NFPAs and is correlated with the development and invasion of NFPAs.

## Background

Pituitary adenomas (PAs) account for up to 15% of intracranial tumors and have a prevalence of 80–90/100,000 people [1,2]. Only approximately 0.2% of PAs with subarachnoid, brain, or systemic metastasis are considered to be malignant [3]. However, many PAs are capable of aggressive growth and invade surrounding structures such as the sphenoidal sinus, cavernous sinus, and third ventricle, and are described as invasive PAs. Curative radical surgery remains difficult for invasive PAs, which tend to recur quickly and even have a fatal outcome [4].

Nonfunctioning pituitary adenomas (NFPAs) consist of approximately 30% of all pituitary tumors, reflecting a very heterogeneous group [5]. As hormonal inactivity leads to delayed diagnosis compared with functioning PAs, NFPAs sometimes present with invasive macroadenomas that cause severe neurological symptoms [6]. There is no effective drug for most cases of NFPAs, while radiotherapy has potential side effects including hypopituitarism, neurocognitive dysfunction, and cerebrovascular disease [6]. Therefore, NFPA therapy remains a challenge for clinicians.

PAs are monoclonal in nature, suggesting that they arise from a primary abnormal pituitary cell that possesses a unique proliferation advantage [7]. Subsequently, additional tumor-promoting factors may confer increased proliferative and aggressive potential to the PA cells. However, the mechanisms of aggressive biological behavior of some PAs have not been fully understood [8,9].

Transforming growth factor β (TGF-β) signaling functions as a suppressor or a promoter in tumor development, depending on the tumor stage and type [10,11]. TGF-β signaling is initiated by the binding of ligands (TGF-β1, TGF-β2, and TGF-β3) to type II TGF-β receptors (TGF-β RII), followed by recruitment of the type I TGF-β receptor (TGF-β RI) to form the complex. Next, TGF-β RII phosphorylates TGF-β RI to activate it. Activated TGF-β RI propagates signaling by phosphorylating Smad2 and Smad3, which then form a heteromeric complex with Smad4 and translocate into the nucleus to regulate gene expression. Smad7 inhibits TGF-β mediated phosphorylation of Smad2 and Smad3, thereby suppressing downstream TGF-β signaling [12-14].

The clinical significance of TGF-β ligands and downstream signaling mediators has been studied in many types of tumors, and the results are discordant [15-18]. Until now, the role of TGF-β signaling in the development and invasion of NFPAs has not been explored. In order to investigate the role of the TGF-β signaling pathway in tumor development, combining several biomarkers of the TGF-β pathway may be superior to the analysis of a single component of the pathway [18]. Accordingly, in this study, we examined the expression of TGF-β1, Smad2, phospho-Smad2 (p-Smad2), Smad3, phospho-Smad3 (p-Smad3), Smad4, and Smad7 in normal anterior pituitaries, invasive NFPAs, and noninvasive NFPAs and evaluated whether they were correlated with tumor development and invasion. Additionally, proliferating cell nuclear antigen (PCNA) was evaluated as an indicator of NFPA proliferation.

## Methods

### Patients and specimens

NFPAs were obtained from 50 patients (23 men and 27 women; 50.9 ± 10.5 years; range, 26–71 years) who underwent endoscopic transsphenoidal surgery between March 2010 and December 2012 at Beijing Tiantan Hospital. Patients who had received previous radiation therapy or had recurrence were not included in this study. The diagnosis of NFPA was confirmed according to clinical manifestation, hormonal and magnetic resonance imaging (MRI) information, as well as histopathological analysis and immunohistochemical staining for all anterior pituitary hormones. Forty-three NFPAs were negative for hormone expression, and seven NFPAs were positive for luteinizing hormone (LH) and/or follicle-stimulating hormone (FSH). NFPAs that were stained positive for hormone expression were termed NF^+^PAs, whereas those negative for hormone expression were considered NF^-^PAs. In addition, five normal human anterior pituitaries were obtained from a donation program. All of the donors died of non-neurological and nonendocrine diseases. The donors consisted of 3 men and 2 women (35.6 ± 9.8 years; range, 32–54 years). Invasive PAs were defined as grade IV based on Hardy-Wilson classification and/or grade III and IV based on Knosp classification [19,20]. There were 29 invasive NFPAs and 21 noninvasive NFPAs. The study protocol was approved by the Ethics Committee of Beijing Tiantan Hospital, and informed consent was obtained from all patients. The patient characteristics are summarized in Table 1.

**Table 1:** Characteristics of patients

	**Invasive NFPAs**	**Noninvasive NFPAs**
Demographics		
Number	29	21
Age (years)	51.9 ± 11.0 (26–68)	49.6 ± 9.9 (29–71)
Male/Female	13 (44.8%)/16 (55.2%)	11 (52.4%)/10 (47.6%)
Symptom		
Headache	20 (69.0%)	10 (47.6%)
Visual deficit	13 (44.8%)	6 (28.6%)
Hypopituitarism	14 (48.3%)	2 ( 9.5%)
None	3 (10.3%)	12 (57.1%)
Classification		
NF+	4 (13.8%)	3 (14.3%)
NF-	25 (86.2%)	18 (85.7%)
Tumor size (cm)		
<3	2 ( 6.9%)	18 (85.8%)
≥3	27 (93.1%)	3 (14.2%)

### Real-time quantitative reverse transcription polymerase chain reaction (qRT-PCR)

NFPAs and normal human anterior pituitaries were stored in liquid nitrogen immediately after harvesting. Total RNA was extracted from the frozen tumor samples and normal anterior pituitaries using Trizol (Invitrogen, Carlsbad, CA, USA) according to the manufacturer’s instructions. The purity and concentration of RNA were determined by a NanoDrop 1000 instrument (Thermo Scientific, Wilmington, DE). The cDNA was synthesized from total RNA (5 μg) using a first-strand cDNA synthesis kit (Invitrogen, Carlsbad, CA, USA) according to the manufacturer’s instructions. qRT-PCR was performed on an ABI 7500 Fast system (Applied Biosystems, Foster City, CA, USA) using the Platinum SYBR Green qPCR SuperMix-UDG kit (Invitrogen, Carlsbad, CA, USA) according to the manufacturer’s instructions. The amplification conditions were 50°C for 2 min, 95°C for 2 min, then 40 cycles at 95°C for 15 s and 60°C for 30 s. GAPDH was used as an internal control. Relative mRNA levels were calculated based on the C_T_ values, corrected for GAPDH expression, according to the equation: 2 ^–ΔCT^ (ΔC_T_ = C_T_ gene of interest – C_T_ GAPDH) [21]. PCR specificity was determined by dissociation curve analysis. All qRT-PCRs were performed in triplicate. The primers used for RT-PCR are listed in Table 2.

**Table 2:** Primers used in this study

**Gene**	**Forward Sequence (5**′**-3**′**)**	**Reverse sequence (5**′**-3**′**)**
Smad2	ATCCTAACAGAACTTCCGCC	CTCAGCAAAAACTTCCCCAC
Smad3	GGAGAAATGGTGCGAGAAGG	GAAGGCGAACTCACACAGC
Smad4	GCATCGACAGAGACATACAG	CAACAGTAACAATAGGGCAG
Smad7	GAATCTTACGGGAAGATCAACCC	CGCAGAGTCGGCTAAGGTG
TGF-β1	CCCTGGACACCAACTATTGC	TGCGGAAGTCAATGTACAGC
PCNA	ACTAACTTTTGCACTGAGGTACC	GTATTTTAAGTGTCCCATATCCGC
GAPDH	GAAGGTCGGAGTCAACGGATT	CGCTCCTGGAAGATGGTGAT

### Western blot analysis

Total protein was extracted from NFPAs or normal pituitaries using lysis buffer (Applygen, Beijing, China) following the manufacturer’s instructions. The protein concentration was measured using the bicinchoninic acid method. Protein samples (30 μg) were separated by electrophoresis using 10% sodium dodecyl sulfate polyacrylamide gels, and then transferred to polyvinylidene difluoride membranes. The membranes were blocked with 5% bovine serum albumin in Tris-buffered saline containing 0.1% Tween 20, and incubated with rabbit antibodies against human Smad2 (1:1000; Cell Signaling Technology, Boston, MA, USA), p-Smad2 (Ser465/467) (1:1000; Cell Signaling Technology, Boston, MA, USA), Smad3 (1:1000; Cell Signaling Technology, Boston, MA, USA), p-Smad3 (Ser423/425) (1:1000; Cell Signaling Technology, Boston, MA, USA), and GAPDH (1:5000; Sigma-Aldrich, St. Louis, MO, USA). Subsequently, the membranes were incubated with the secondary antibody anti-rabbit IgG (1:4000; Jackson ImmunoResearch Laboratories, West Grove, PA, USA). Finally, the membranes were developed using an ultrasensitive chemiluminescence protein dye detection system (ECL Plus; Amersham Pharmacia Biotech, Piscataway, NJ, USA) and exposed to X-ray films (Kodak, Rochester, NY, USA). The bands were subjected to grayscale scanning and semi-quantitative analysis using Quantity One software (Bio-Rad, Hercules, CA, USA).

### Immunohistochemistry

The tissues were fixed in 10% buffered formalin and embedded in paraffin. Paraffin-embedded tissues were cut into 5-μm serial sections, then transferred onto adhesive slides, and dried at 65°C for 30 min. The sections were deparaffinized with xylene and rehydrated through graded alcohol soluntions. Endogenous peroxidase activity was blocked with 3% hydrogen peroxide solution for 30 min, and antigen retrieval was performed at 100°C for 20 min in citrate buffer (10 mM, pH 6.0). After washing three times with phosphate-buffered saline (PBS) for 5 min each, the sections were incubated with 10% normal goat serum to block nonspecific binding. Then, the sections were incubated with Smad3 (1:100; Abcam, Cambridge, MA, USA) or p-Smad3 (Ser423/425) (1:100; Abcam, Cambridge, MA, USA) antibody at 4°C overnight followed by immunodetection using the two-step polymer detection system (Polink-2 plus Kit; GBI Labs, Manchester, England) and visualized with 3,3′-diaminobenzidine. The slides were counterstained with Mayer’s hematoxylin, dehydrated in graded alcohol, and mounted with a neutral resin. For a negative control, the primary antibody was replaced with PBS.

All sections were examined and scored by both two pathologists without knowledge of the patient’s clinical record. Five fields at 400× magnification were randomly selected. Staining scores for Smad3 and p-Smad3 were determined by a semi-quantitative system, using the percentage of positive cells and staining intensity from 1 to 3 (1, weak; 2, moderate; and 3, strong), and the immunostaining results were classified according to the scores as negative (0–30), weakly positive (31–150), or strongly positive (150–300) [22].

### Statistical analysis

Statistical analysis was performed with SPSS v16.0 software (IBM Corporation, Armonk, NY, USA). The data were expressed as mean ± standard deviation. Differences between groups were determined by one-way analysis of variance and the two independent samples t-test or the Mann–Whitney U test. The correlation analysis was assessed by Spearman’s rank correlation. *P* < 0.05 was defined as statistically significant.

## Results

### Comparison of Smad3 and p-Smad3 levels in invasive NFPAs and noninvasive NFPAs

**Figure 1:**
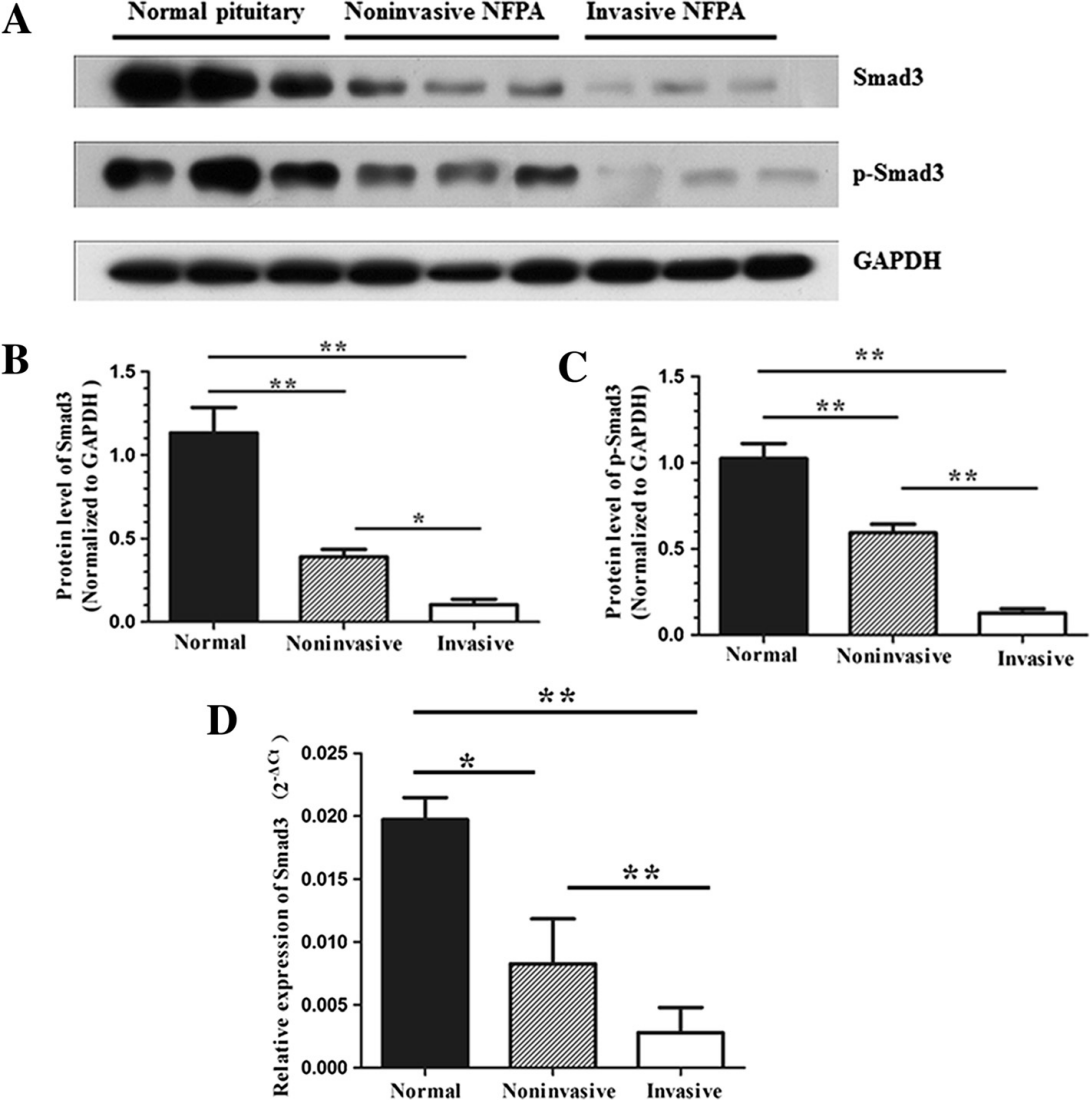
**Western blot and qRT-PCR analyses of Smad3 and p-Smad3. (A, B and C)** Western blot and densitometric analyses of Smad3 and p-Smad3. **(D)** qRT-PCR analysis of Smad3. *, *P* < 0.05; **, *P* < 0.01.

Western blot analysis demonstrated that the Smad3 protein level was significantly less in invasive NFPAs (*P* < 0.01) and noninvasive NFPAs (*P* < 0.01) compared to normal anterior pituitaries (Figure 1A and B). In addition, the Smad3 protein level was significantly less in invasive NFPAs compared to noninvasive NFPAs (*P* < 0.05) (Figure 1A and B).

Next, we detected Smad3 mRNA expression by qRT-PCR. The Smad3 mRNA level was significantly less in invasive NFPAs (*P* < 0.01) and noninvasive NFPAs (*P* < 0.05) compared to normal anterior pituitaries (Figure 1D). Moreover**,** Smad3 mRNA level was significantly less in invasive NFPAs compared to noninvasive NFPAs (*P* < 0.01) (Figure 1D). The correlation coefficient between Smad3 mRNA and protein was 0.836 (*P* < 0.05), suggesting a strong correlation between Smad3 mRNA and protein levels.

**Figure 2:**
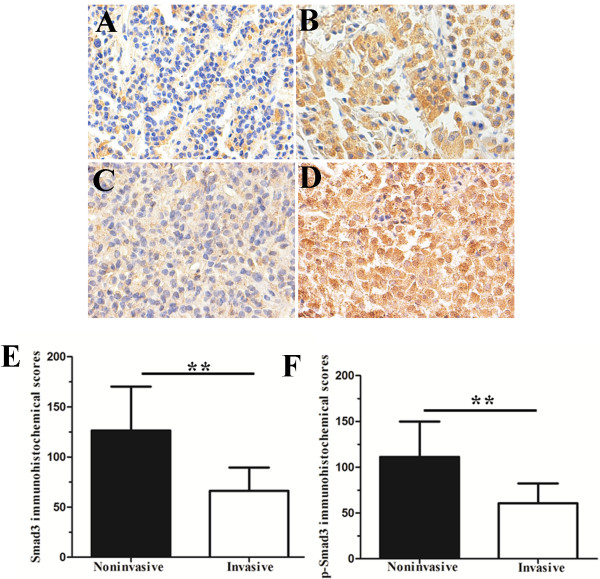
**Immunohistochemical analysis of Smad3 and p-Smad3. (A)** Low Smad3 expression in invasive NFPA (case 1). **(B)** High Smad3 expression in noninvasive NFPA (case 2). **(C)** Low p-Smad3 expression in invasive NFPA (case 1). **(D)** High p-Smad3 expression in noninvasive NFPA (case 2). **(E and F)** The staining scores of Smad3 and p-Smad3. Magnification: 400×. **, *P* < 0.01.

By immunohistochemical analysis, Smad3 and p-Smad3 protein were detected in nearly all anterior pituitary cells. The Smad3 level was significantly less in invasive NFPAs compared to noninvasive NFPAs (*P* < 0.01) (Figure 2A, B and E). Smad3-positive staining was observed predominantly at the cell membrane and/or in the cytoplasm, as well as in the nucleus.

For determination of the activated Smad3 (p-Smad3) protein level, western blot analysis showed that the p-Smad3 protein level was significantly less in invasive NFPAs (*P* < 0.01) and noninvasive NFPAs (*P* < 0.01) compared to normal anterior pituitaries (Figure 1A and C). In addition, the p-Smad3 protein level was significantly less in invasive NFPAs compared to noninvasive NFPAs (*P* < 0.01) (Figure 1A and C). Immunohistochemical analysis confirmed that the p-Smad3 level was significantly less in invasive NFPAs compared to noninvasive NFPAs (*P* < 0.01) (Figure 2C, D, and F). p-Smad3-positive staining was observed predominantly in the nucleus, as well as at the membrane and/or in the cytoplasm.

### Comparison of Smad2 and p-Smad2 levels in invasive NFPAs and noninvasive NFPAs

**Figure 3:**
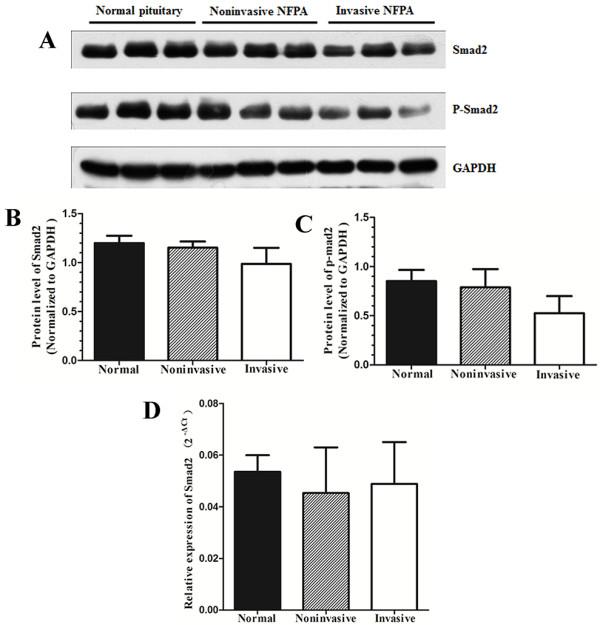
**Western blot and qRT-PCR analyses of Smad2 and p-Smad2. (A, B and C)** Western blot and densitometric analyses of Smad2 and p-Smad2. **(D)** qRT-PCR analysis of Smad2.

By Western blot and qRT-PCR analyses, we found no significant difference in Smad2 (*P* = 0.122) or p-Smad2 protein levels (*P* = 0.101) (Figure 3) or the Smad2 mRNA level in normal pituitary tissues, invasive NFPAs, and noninvasive NFPAs (*P* = 0.409) (Figure 3).

### Comparison of TGF-β1, Smad7, and Smad4 levels in invasive NFPAs and noninvasive NFPAs

**Figure 4:**
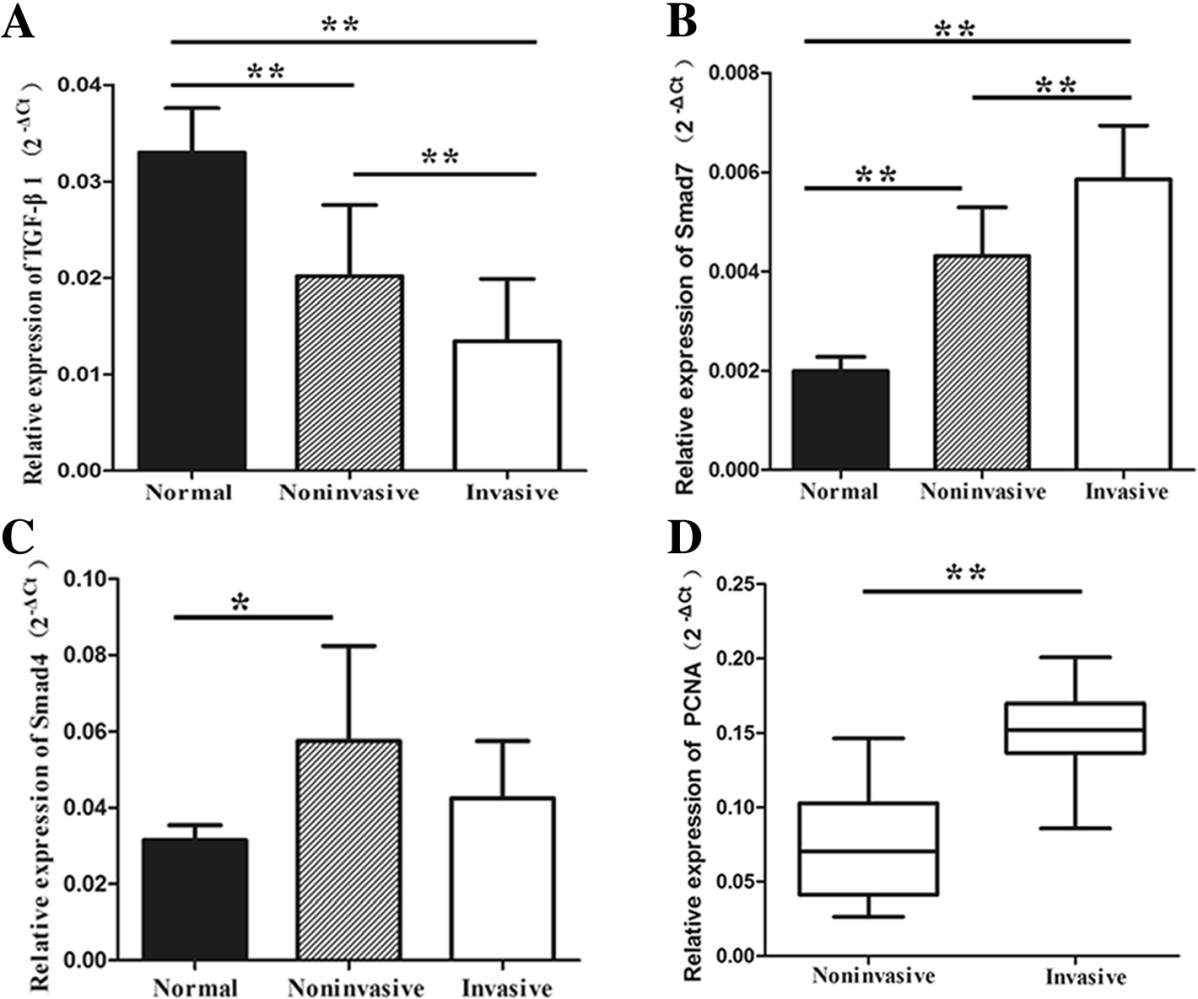
**qRT-PCR analysis of TGF-β1, Smad7, Smad4 and PCNA. (A)** The mRNA expression of TGF-β1. **(B)** The mRNA expression of Smad7. **(C)** The mRNA expression of Smad4. **(D)** The mRNA expression of PCNA. *, *P* < 0.05; **, *P* < 0.01.

qRT-PCR analysis showed that the TGF-β1 mRNA level was significantly less in invasive NFPAs (*P* < 0.01) and noninvasive NFPAs (*P* < 0.01) compared to normal anterior pituitaries (Figure 4A). In addition, the TGF-β1 mRNA level was significantly less in invasive NFPAs compared to noninvasive NFPAs (*P* < 0.01) (Figure 4A).

On the contrary, the Smad7 mRNA level was significantly greater in invasive NFPAs (*P* < 0.01) and noninvasive NFPAs (*P* < 0.01) than in normal anterior pituitaries (Figure 4B). Moreover, the Smad7 mRNA level was greater in invasive NFPAs than in noninvasive ones (*P* < 0.01) (Figure 4B).

The Smad4 mRNA level was greater in noninvasive NFPAs than in normal anterior pituitaries (*P* < 0.05) (Figure 4C). However, there was no difference in the expression of Smad4 mRNA between invasive and noninvasive NFPAs (*P* = 0.076) (Figure 4C). In addition, there was no significant difference in the Smad4 mRNA level between invasive NFPAs and normal anterior pituitaries (*P* = 0.897) (Figure 4C).

As an indicator for the proliferation activity of tumor cells, PCNA was used to evaluate tumor growth. qRT-PCR analysis showed that the PCNA mRNA level was markedly greater in invasive NFPAs compared to noninvasive ones (*P* < 0.01) (Figure 4D). By Spearman’s rank correlation analysis, the PCNA mRNA level showed a significant correlation with the Smad3 mRNA level (*P* < 0.01, r = -0.697). However, the PCNA mRNA level was not significantly correlated with the level of Smad2 (*P* = 0.232), Smad4 (P = 0.263), Smad7 (*P* = 0.242), or TGF-β1 (*P* = 0.171).

## Discussion

The TGF-β signaling pathway is involved in a diverse set of cellular processes, such as cell proliferation, differentiation, migration, apoptosis, and biological processes including embryonic development, immunity regulation, and tissue homeostasis [23-25]. The role of TGF-β signaling in tumorigenesis is complex. In normal epithelial cells, TGF-β acts as a potent tumor suppressor and prevents incipient tumors from progression to malignancy [11]. However, due to subsequent inactivation of TGF-β signaling or key target genes, malignant cells will lose TGF-β tumor-suppressive responses. In addition, pathological forms of TGF-β signaling can promote tumor growth and invasion, the evasion of immune surveillance, and tumor cell dissemination and metastasis [11].

Several studies have shown the tumor suppressor role of Smad3, whose deficiency contributes to tumor formation and development [26,27]. Consistently, we found that a low Smad3 or p-Smad3 protein level and a low Smad3 mRNA level were closely associated with NFPA development and invasion. Smad7 is an inhibitory Smad, which can suppress TGF-β-mediated phosphorylation of Smad2 and Smad3 as well as prevent their interaction with Smad4 and subsequent nuclear translocation. Kleeff et al. have demonstrated that Smad7 enhances tumorigenicity in pancreatic cancer [28]. Moreover, Halder et al. have reported that Smad7 induces hepatic metastasis in colorectal cancer [29]. In this study, we found that the expression of Smad7 mRNA increased gradually from normal anterior pituitaries, noninvasive NFPAs, to invasive NFPAs, implying that the upregulation of Smad7 contributes to NFPA development. These data suggest that the balance between Smad3 and Smad7 may affect the development and invasion of NFPAs.

Smad4 was originally identified as a tumor suppressor gene in pancreatic carcinomas [30]. Subsequently, many studies have shown that Smad4 is underexpressed in various human tumors, including stomach cancer, squamous cell carcinoma of the esophagus, and breast cancer, and Smad4 has been proposed as a prognostic marker for tumor formation and progression [18,31,32]. Surprisingly, in the present study, we found that the Smad4 mRNA level was greater in noninvasive NFPAs than in normal anterior pituitaries. In addition, the difference in the Smad4 mRNA level between invasive NFPAs and normal anterior pituitaries was not significant. Moreover, there was no significant difference in the Smad4 mRNA level between invasive and noninvasive NFPAs. These results suggest that Smad4 may not act as a tumor suppressor in NFPAs, but further studies are needed to confirm our speculation.

Since TGF-β1 is upregulated to a greater extent than either TGF-β2 or TGF-β3 in cancer, TGF-β1 has been the focus for cancer researchers [33]. Interestingly, we found that the TGF-β1 mRNA level gradually decreased from normal anterior pituitaries, noninvasive NFPAs, to invasive NFPAs. These data indicate that TGF-β1 may be a suppressor of NFPA development and invasion. It has been shown that the thrombospondin-1 analogs ABT-510 and ABT-898 increased the activation of TGF-β1 in the pituitary, possibly contributing to the inhibition of prolactinoma growth [34].

Additionally, we evaluated the expression of PCNA mRNA and found that increased PCNA mRNA expression was associated with NFPA invasiveness. PCNA is a nuclear protein that has been identified as the auxiliary protein of deoxyribonucleic acid polymerase delta, and its expression level is correlated with cell proliferation [35]. A previous study has reported that the PCNA level was greater in macroadenomas than in microadenomas and that a higher PCNA index was correlated with a shorter disease-free interval [36]. Furthermore, we found that the PCNA level was negatively correlated to the Smad3 level, suggesting that downregulation of Smad3 may contribute to the inactivation of TGF-β signaling and the promotion of tumor growth.

To the best of our knowledge, this is the first study to systematically investigate the differential expression of TGF-β signaling pathway components in the normal pituitary, invasive NFPAs, and noninvasive NFPAs. We hypothesized that the activity of TGF-β signaling is restrained and that the role of TGF-β in tumor suppression is impaired in NFPAs. Thus, NFPAs have not circumvented the suppressive effects of the TGF-β signaling pathway. Consequently, recovering the capability of TGF-β signaling to suppress tumor development is a promising therapeutic strategy for NFPAs, especially for invasive NFPAs.

Furthermore, our data suggest that Smad3 and p-Smad3 may be used as significant biomarkers to identify invasive NFPAs in clinical practice. So far, only a few biomarkers have been proposed to discern invasive NFPAs. By immunohistochemical and western blot analyses, we found that Smad3 and p-Smad3 levels were negatively correlated with tumor invasion. Consequently, Smad3 and p-Smad3 may be useful biomarkers for the diagnosis of invasive NFPAs.

However, there were several limitations of our study. Firstly, due to the small sample size in our study, there may be some selection bias, and we think that it is necessary to confirm our conclusion in further study with a large sample size. In addition, active TGF-β1, which is relative to the total [37], is very low. It was not taken into account because tumor samples we obtained were few and they were not uniformly available across studies.

In summary, this study demonstrated that a low Smad3 or p-Smad3 protein level was closely associated with NFPA development and invasion. Moreover, the low expression of Smad3 and TGF-β1 mRNA and the high expression of Smad7 mRNA were associated with NFPA development and invasion. These data suggest that the TGF-β signaling pathway plays an important role in the progression and invasion of NFPAs and promises to be a new target for the diagnosis and treatment of NFPAs.

## Abbreviations

PA: Pituitary adenoma; NFPA: Nonfunctioning pituitary adenomas; TGF-β: Transforming growth factor β; TGF-β RII: Type II TGF-β receptors; TGF-β RI: Type I TGF-β receptor; p-Smad2: Phospho-Smad2; p-Smad3: Phospho-Smad3; PCNA: Proliferating cell nuclear antigen; MRI: Magnetic resonance imaging; LH: Luteinizing hormone; FSH: Follicle-stimulating hormone; qRT-PCR: Real-time quantitative reverse transcription polymerase chain reaction.

## Competing interests

The authors declare that they have no conflict of interest.

## Authors’ contributions

LZ, LC and ZY participated in the study design and wrote the paper. LZ, WY, HL, CL and WH carried out the experimental studies. LX, WY and WF participated in manuscript revision. All authors read and approved the final manuscript.
